# Diagnostic Accuracy of Waist-to-Height Ratio, Waist Circumference, and Body Mass Index in Identifying Metabolic Syndrome and Its Components in Older Adults: A Systematic Review and Meta-Analysis

**DOI:** 10.1016/j.cdnut.2023.102061

**Published:** 2023-12-12

**Authors:** Vicky Chan, Liujiao Cao, Martin Ming Him Wong, Kenneth Lo, Wilson Tam

**Affiliations:** 1 Department of Food Science and Nutrition, The Hong Kong Polytechnic University, Hong Kong, China; 2 West China School of Nursing/West China Hospital, Sichuan University, Chengdu, China; 3 Institute of Epidemiology and Health Care, University College London, London, United Kingdom; 4 Research Institute for Smart Ageing, The Hong Kong Polytechnic University, Hong Kong, China; 5 Alice Lee Centre for Nursing Studies, Yong Loo Lin School of Medicine, National University of Singapore, Singapore

**Keywords:** metabolic syndrome, older adults, receiver operating characteristic curve, waist-to-height ratio

## Abstract

**Background:**

Although numerous studies have indicated the utility of waist-to-height ratio (WHtR) in early screening for individuals with adverse cardiometabolic health, there is controversy on using WHtR as a one-size-fits-all approach, including in older adults.

**Objectives:**

Our study aims to identify the pooled diagnostic accuracy of WHtR in screening for metabolic syndrome (MetS) and its components among older adults.

**Methods:**

A systematic review of observational studies was performed using 4 databases. A diagnostic meta-analysis with a random effects model was conducted, and the pooled area under the summary receiver operating characteristic curve, sensitivity, specificity, positive and negative likelihood ratios, and diagnostic odds ratio (dOR) of each outcome compared with WHtR, body mass index (BMI), and waist circumference (WC) were calculated, with sex-stratified analysis.

**Results:**

A total of 17 studies with 74,520 participants were included. As reflected by the dOR, WHtR (7.65; 95% CI: 6.00, 9.75) performed better than BMI (5.17; 95% CI: 4.75, 5.62) and WC (5.77; 95% CI: 4.60, 7.25) in screening for MetS among older adults and was potentially better among males. For hyperglycemia, hypertension, and dyslipidemia, the performances of WHtR, BMI, and WC were comparable.

**Conclusion:**

More studies focusing on older adults are still needed to determine the cutoff values of WHtR to screen for MetS.

The search strategy was registered in PROSPERO as CRD42022350379.

## Introduction

Metabolic syndrome (MetS) describes a group of metabolic abnormalities including elevated blood pressure and blood glucose, abdominal obesity, and high triglycerides and low HDL levels in the blood [1]. A previous meta-analysis demonstrated the possible association between MetS and higher risk of cardiovascular diseases (CVDs) [2], which constitute a significant global health burden. Another 6.5-y cohort study with 5110 participants reported that when compared to people without MetS, individuals with MetS more often had diabetes, hypertension, and CVD, as well as a higher risk of all-cause mortality (hazard ratio [HR]: 1.26; 95% confidence interval [CI]: 1.04, 1.54) and cardiovascular events (HR: 1.48, 95% CI: 1.22, 1.79) [3]. A previous systematic analysis studying the global health burden of CVDs in 195 countries revealed that from 1990 to 2017, the number of deaths due to CVDs increased by 48.62% [4]. Another study reported that in 2017, 6.28% people in the world had type 2 diabetes, and more than one million deaths were caused by diabetes each year [5]. Hence, valid and easy-to-measure indicators are required for screening for MetS, which would allow for early detection and prevention of cardiometabolic diseases.

Although BMI is a simple and widely used anthropometric index, accumulating evidence from prospective studies suggests that obesity indices such as waist-to-height ratio (WHtR) and waist circumference (WC) have better predictive power for diagnosing MetS [[6], [7], [8]]. A pooled analysis of prospective cohorts showed that a higher WC is associated with higher all-cause and CVD mortality regardless of BMI category [9,10]. Results from the Nurses’ Health Study also confirmed that elevated WC was positively associated with all-cause and CVD mortality independent of BMI [11]. When compared to WC, WHtR is potentially a better screening tool because it accounts for variations in height [12], and it can be easily derived from WC and BMI data without further assessment. Receiver operating characteristic curves from a meta-analysis showed that WHtR had better screening power (as reflected by the pooled area under the curve [AUC]) than WC in classifying diabetes, hypertension, and CVD among adults, regardless of sex [13]. Furthermore, elevated WHtR was associated with a higher risk of diabetes [14] and CVD morbidity [15] in cohort studies. Given the utility of WHtR in early health screening, the National Institute for Health and Care Excellence plans to update their clinical guideline [16], encouraging individuals to assess WHtR instead of WC to identify central obesity and for people with elevated WHtR to seek medical advice for further clinical assessment.

Although numerous studies have indicated the utility of WHtR in early screening for individuals with adverse cardiometabolic health, there is controversy on using WHtR for health screening in a one-size-fits-all approach. For example, a meta-analysis examined the discriminatory power of WHtR to detect elevated cardiometabolic risk in children and adolescents [17] and found that WHtR did not have significantly better screening power than 2 other indexes in most outcomes, except for elevated triglycerides, when compared with BMI and high metabolic risk score when compared with WC [17]. The authors postulated that the rapid change in height from childhood to adolescence outweighs increases in WC, which may misclassify fast-growing children as being healthy despite having excess abdominal fat [17].

Moreover, identifying risk of MetS in older adults also warrants more attention. When using WHtR to identify cardiometabolic risk, the proportion of individuals with WHtR ≥0.5 increased systematically for people with shorter stature [18], which is more prevalent in older adults that tend to lose height due to osteoporosis [19]. In other words, using WHtR ≥0.5 as a threshold value may misclassify older adults with elevated cardiometabolic risk. According to the World Health Organization, the world’s older population will double from 12% to 22% by 2050 [20]. Aging is one of risk factors leading to MetS, which has a significant association with mortality in the older people [21]. However, there has been no systematic approach to determine the cutoff values of WHtR to screen for MetS among older adults and measure its discriminatory power when compared to other conventional indicators, namely BMI and WC. Hence, our study aims to compare the diagnostic accuracy of WHtR with that of BMI and WC in screening older adults for MetS and its components.

## Methods

### Search strategy

The literature search complied with PRISMA guidelines [22]. A comprehensive literature search was performed in 4 databases, including Embase via Ovid (1910 to Present); Ovid Emcare (1995 to 2022 Week 29); Ovid MEDLINE and Epub Ahead of Print, In-Process, In-Data-Review & Other Non-Indexed Citations (1946 to July 28, 2022); and Ovid Nursing Database (1946 to July Week 4 2022). The following search terms were used: *1*) waist-to-height OR waist to height OR waist height OR waist to ht OR waist-to-ht OR waist ht OR wst height OR wst ht OR WHtR OR waist circumference to height OR waist stature OR waist-stature OR waist-to-stature OR WSR AND *2*) older adult∗ OR older people OR older person∗ OR elder∗ OR aged AND *3*) sensitiv∗ OR specific∗ OR diagnostic accuracy OR Receiver operating characteristic OR ROC OR true positive∗ OR false positive∗ OR true negative∗ OR false negative∗ OR area under the curve OR AUC OR AUSROC OR likelihood ratio OR Positive likelihood ratio OR PLR OR Negative likelihood ratio OR NLR OR predictive value∗ OR Positive predictive value OR PPV OR Negative predictive value OR NPV OR diagnostic odds ratio OR DOR OR diagnosis OR screening OR detection. Articles with English abstracts were assessed. The search strategy was registered in PROSPERO (CRD42022350379).

### Study inclusion criteria

1. Observational studies (i.e., prospective or retrospective, and cross-sectional studies).
2. Adults aged ≥65 y (or mean age ≥65 y) or studies of the general population that provided data on a subgroup of older adults.
3. Studies that contained data on WHtR measurement.
4. At least one biomarker or a cluster of them as the primary outcome related to MetS (elevated fasting blood glucose, elevated blood pressure, dyslipidemia, elevated total cholesterol, elevated triglyceride, low HDL cholesterol, elevated LDL cholesterol, and clustered cardiometabolic risk [MetS or the sum of risk factors]).
5. Studies that stated sensitivity, specificity, AUC, negative predictive value, positive predictive value, or other data related to diagnostic accuracy.
6. Articles with full text in the English language in peer-reviewed journals.

### Study exclusion criteria

1. Studies conducted only in populations with overweight or obesity.
2. Studies that did not calculate the optimal WHtR cutoff.
3. Studies that did not report the prevalence of any cardiometabolic risk factor.
4. Studies based on data from the same surveys or studies to avoid duplicate data, and studies with larger sample sizes.
5. Intervention studies, review papers, protocols, editorials, abstracts, or congress communications.

### Study selection

Deduplication was performed during the Ovid database search. After removing duplicated studies, 2 reviewers (KL and LC) independently performed initial screening of the titles and abstracts with the inclusion and exclusion criteria listed above. Full texts of potential articles were assessed for eligibility by independent reviewers (LC and MW). Any discrepancies between 2 reviewers were solved by group discussion with all authors.

### Data extraction and quality assessments

For each included study, study characteristics including first author, publication year, study location, sample size, study design, age and sex of participants, exposure variables (i.e., BMI, WC, WHtR), and outcomes and definitions of MetS or its components (e.g., dyslipidemia, hypertension, and hyperglycemia) were independently extracted by 2 reviewers (KL and LC) using a standardized data extraction form. Accuracy indicators (i.e., sensitivity, specificity, true positive [TP], false positive [FP], true negative [TN], and false negative [FN]) were also extracted. Quality assessment of all included studies was conducted by LC and MW independently using the Quality Assessment of Diagnostic Accuracy Studies-2 (QUADAS-2) tool [23]. A total of 4 domains were included: patient selection, index test, reference standard, and flow and timing. All domains were evaluated in terms of risk of bias, and the first 3 domains were also evaluated in terms of applicability. Risk of bias was judged as ‘low,’ ‘high,’ or ‘unclear.’ Any disagreement between the 2 authors (LC and MW) was resolved by group discussion with all authors.

### Statistical analysis

Meta-DiSc 2.0, a web-based software (https://ciberisciii.shinyapps.io/MetaDiSc2/), was used for the diagnostic meta-analysis with a bivariate random effects model [24] that estimates the summary of sensitivity and specificity, acknowledging any possible (negative) correlation between these 2 measures that, in turn, partially addresses the threshold effect.

The area under the summary receiver operating characteristic (AUSROC) curve, sensitivity, specificity, positive likelihood ratio, negative likelihood ratio, correlation between the sensitivities and FP rates, and diagnostic odds ratio (dOR), as well as their corresponding 95% confidence intervals (CI), were computed using the extracted TP, FP, TN, and FN data from each included study.

Summary receiver operating characteristic (SROC) curves were created to illustrate the pooled sensitivity and specificity of each cardiometabolic outcome with different exposures. To investigate the heterogeneity, the bivariate *I*^2^ statistic was computed, and heterogeneity was described as moderate (*I*^2^ >30%), substantial (*I*^2^ >50%) or considerable (*I*^2^ >75%) [25]. Subgroup analysis by sex was also conducted for outcomes with ≥5 studies to evaluate the accuracy of different exposures in identifying cardiometabolic outcomes.

## Results

### Study selection

**Figure 1:**
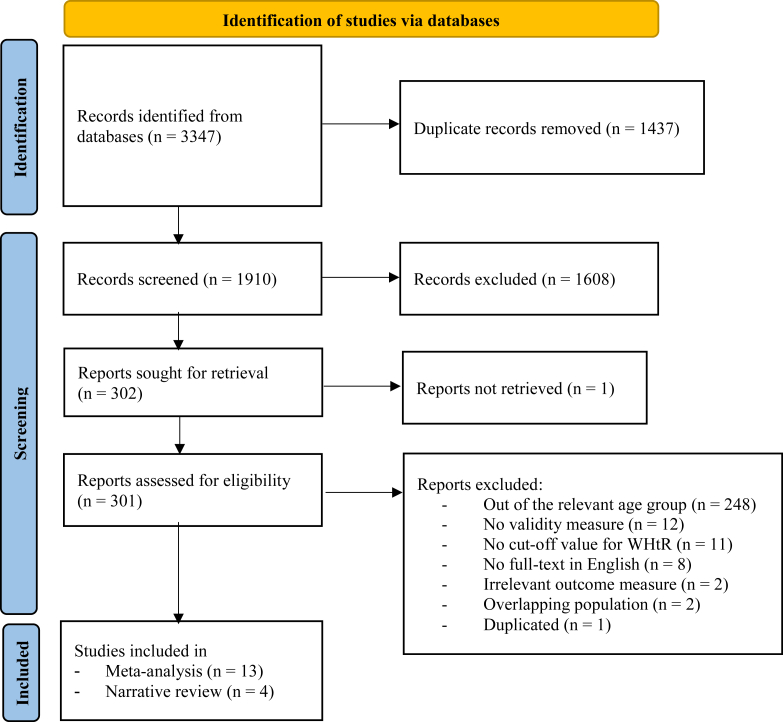
Flow diagram of study selection.

A total of 1910 articles were initially identified from the database search after the removal of duplicates. After title and abstract screening, the full texts of 301 articles were reviewed for eligibility. Finally, after full-text evaluation, a total of 17 studies were included. Of these, 13 studies were included for meta-analysis while the remaining 4 studies were presented narratively. The study selection summary and reasons for exclusion are presented in the PRISMA flowchart in Figure 1.

### Included studies

The study characteristics and outcome definitions of 17 included studies with a total of 74,520 participants are shown in Table 1 [[26], [27], [28], [29], [30], [31], [32], [33], [34], [35], [36], [37], [38], [39], [40], [41], [42]]. All studies were published from 2014 to 2021. Four studies were conducted in Brazil [26,35,37,39], 5 in China [28,30,33,34,42], 1 in Colombia [40], 2 in Iran [27,36], 2 in Japan [31,32], 1 in Singapore [41], 1 in Spain [29], and 1 in Thailand [38]. Sample sizes ranged from 159 to 24,215 participants. The diagnostic accuracies of all included studies are summarized in Table 2 [43]. As summarized in Supplemental Table 1, the optimal WHtR values of studies included in meta-analysis were mostly within the range of 0.50 and 0.59; the few exceptions were Rodríguez-Guerrero et al. [29] and Ramírez-Vélez et al. [40], who observed WHtR was optimal in screening for MetS in overall or female population, and Ke et al. [33], who found the optimal WHtR to be 0.49 for screening hyperglycemia. However, the number of included studies was inadequate to conduct subgroup analysis by optimal WHtR values.

**TABLE 1:** Study characteristics of the included studies

Authors, year	Country	Study design	Sample size (% male)	Average age or age range	Exposure(s)	Outcome(s)	Outcome(s) definition
Alves et al., 2021 [26]	Brazil	Cross-sectional study	159 (49.7%)	70.9 ± 7.4	WHtR, WC, BMI	MetS	Harmonized MetS criteria, i.e., ≥3 of the 4 following components: 1. WC (for South American populations) ≥80 cm females and ≥90 cm for males; 2. Dyslipidemia (males: HDL-C ≤40 mg/dL and females: ≤50 mg/dL or TG ≥150 mg/dL, or specific treatment); 3. BP (systolic BP ≥130 mmHg or diastolic BP ≥85 mmHg, or specific treatment); 4. Glycemia (≥100 mg/dL, or specific treatment).
Gharipour et al., 2014 [27]	Iran	Cross-sectional study	206 (100%)	71.85 ± 5.44	WHtR, WC, BMI	MetS	The NCEP-ATPIII definition of MetS was met when ≥3 of the following criteria were present: 1. WC ≥102 cm; 2. HDL <40 mg/dL or specific treatment for this lipid abnormality; 3. Triglycerides ≥150 mg/dL or specific treatment for this lipid abnormality; 4. SBP ≥130 mmHg or DBP ≥85 mmHg or treatment of previously diagnosed hypertension; 5. Fasting glucose ≥ 100 mg/dL.
Gu et al., 2018 [28]	China	Cross-sectional study	6722 (45.8%)	With MetS: 70.02 ± 7.21 (male), 70.59 ± 7.51 (female) Without MetS: 69.98 ± 7.42 (male), 69.70 ± 7.75 (female)	WHtR, BMI	MetS	MetS was defined as the presence of ≥3 of those following features: 1. Central obesity: WC ≥90 cm for males or ≥85 cm for females; 2. Elevated BP: systolic blood pressure ≥130 mmHg or diastolic blood pressure ≥85 mmHg, or ongoing antihypertensive medications; 3. Elevated FPG: FPG ≥5.6 mmol/L, or ongoing antidiabetic treatment; 4. Elevated TG: TG ≥1.7 mmol/L; 5. Reduced HDL-C: HDL-C <1.0 mmol/L in males and <1.3 mmol/L in females.
Rodríguez-Guerrero et al., 2020 [29]	Spain	Cross-sectional study	361 (46.8%)	73.2 ± 6.4	WHtR, WC, BMI	MetS	Based on the harmonized definition, i.e., fulfilling 3 of the following 5 criteria: 1. BP ≥130/85 mmHg or being under antihypertensive treatment; 2. TG ≥150 mg/dL or under treatment with fenofibrate or nicotinic acid; 3. HDL-C <50 mg/dL for females and <40 mg/dL for males, or under treatment with fenofibrate or nicotinic acid; 4. Fasting basal glucose ≥100 mg/dL, under hypoglycemic treatment, or with a diagnosis of T2DM; 5. WC ≥88 cm in females and ≥102 in males (European population cutoff points).
Jiang et al., 2016 [30]	China	Cross-sectional survey	2890 (43.8%)	≥65	WHtR, WC, BMI	Hypertension	Participants were categorized as normotension, prehypertension, and hypertension in terms of SBP and DBP levels set by the JNC-7: 1. Normotension was defined as SBP <120 mmHg and DBP <80 mmHg; 2. Prehypertension was defined as 120 ≤ SBP < 140 mmHg and/or 80 ≤ DBP < 90 mmHg; 3. Hypertension was defined as SBP ≥140 mmHg and/or DBP ≥90 mmHg.
Kawamoto et al., 2019 [31]	Japan	Cross-sectional study	1639 (43.9%)	71 ± 8 (males), 71 ± 7 (females)	WHtR	MetS	Based on the modified criteria of NCEP-ATP III report, MetS was defined as subjects having ≥3 of the following 5 conditions: 1. Abdominal obesity of WC ≥85 cm (males) and ≥80 cm (females) based on the adjusted WC criteria in Japan; 2. High BP (SBP ≥130 mmHg and/or DBP ≥85 mmHg), and/or drug treatment for elevated BP; 3. Hypertriglyceridemia with a TG level ≥150 mg/dL; 4. Low HDL cholesterolemia with an HDL-C <40 mg/dL for males and <50 mg/dL for females, and/or drug treatment for dyslipidemia; 5. High fasting glucose with a HbA1c ≥5.6% (comparable to FPG level ≥100 mg/dL in this study) and/or drug treatment for elevated blood sugar.
Kawamoto et al., 2020 [32]	Japan	Cross-sectional study	1727 (44.5%)	66 ± 9 (Normotension), 72 ± 8 (Hypertension)	WHtR	Hypertension	Hypertension was defined according to the JNC-7 definitions: 1. Being on antihypertensive medication; 2. SBP ≥140 mmHg; 3. DBP ≥90 mmHg.
Ke et al., 2021 [33]	China	Cross-sectional study	24,215 (44.02%)	71.03 ± 5.71	WHtR, WC	T2DM	"With T2DM” was defined as: 1. FPG ≥7.0 mmol/L; 2. Previous diagnosis of T2DM; Using antidiabetic medications.
Liu et al., 2019 [34]	China	Prospective cohort study	4416 (41.2% in total sample)	≥65	WHtR, WC, BMI	Dyslipidemia, Abnormal BP, Hyperglycemia	Dyslipidemia (2016 Chinese guidelines for the management of dyslipidemia in adults): 1. Total cholesterol ≥5.2 mmol/L; 2. LDL-C ≥3.4 mmol/L; 3. HDL-C ≤1.0 mmol/L; 4. TG ≥1.7 mmol/L. Abnormal BP (2010 Chinese guidelines for the management of hypertension): 1. Self-reported hypertension; 2. SBP ≥120 mm Hg; 3. DBP ≥80 mm Hg. Hyperglycemia (WHO criteria): 1. Self-reported diabetes; Fasting blood glucose level ≥5.6 mmol/L.
Vidal Martins et al., 2015 [35]	Brazil	Cross-sectional study	349 (30.95%)	71 (males), 72 (females)	WHtR	Dyslipidemia	Cardiovascular disease risk was calculated by the relation of TG levels with HDL-C levels.
Marzban et al., 2022 [36]	Iran	Prospective cohort study	3000 (48.5%)	67.75 ± 7.10	WHtR, WC, BMI	MetS	MetS was defined as according to the NCEP-ATP III: 1. High BP (elevated BP): ≥130/85 mmHg or known treatment for hypertension; 2. Hypertriglyceridemia (high TG concentration): TG ≥150 mg/dL; 3. Low HDL-C: <40 and < 50 mg/dL in males and females, respectively; 4. Hyperglycemia (high glucose concentration): FBG ≥100 mg/dL or known treatment for diabetes; World Health Organization-Asian Pacific Region criteria for central obesity: WC ≥90 and ≥80 cm in males and females, respectively.
Morais et al., 2018 [37]	Brazil	Cross-sectional study	402 (39.6%)	72.8 ± 7.0 (males), 72.8 ± 7.0 (females)	WHtR, BMI	MetS	Elderly individuals were classified as syndromic according to the Joint Interim Statement (JIS) harmonizing criteria.
Nguyen Ngoc et al., 2019 [38]	Thailand	Cross-sectional survey	15,842 (47.4%)	59.3 ± 13.2	WHtR, WC, BMI	Hypertension	Individuals were diagnosed with hypertension: 1. High blood pressure of SBP ≥140 mmHg or DBP ≥90 mmHg; Used antihypertensive medication during the past 2 wk.
de Oliveira et al., 2016 [39]	Brazil	Cross-sectional study	203 (22.2%)	80.2 ± 9.0	WHtR, WC	MetS	The MetS diagnosis was established based on the MetS-harmonized criterion, so the individual should present a minimum of 3 of the other 4: 1. WC ≥80 cm for females and ≥90 cm for males; 2. Dyslipidemia (HDL-C ≤40 for males and ≤50 for females or specific treatment or TG ≥150 mg/dL or specific treatment); 3. BP (SBP ≥130 mmHg or DBP ≥85 mmHg or specific treatment); Glycemia (≥100 mg/dL or specific treatment).
Ramírez-Vélez et al., 2019 [40]	Colombia	Cross-sectional study	1502 (39.7%)	70 ± 7.6	WHtR, BMI	MetS	MetS was defined according to the most recent Joint Interim Statement of the IDF by adopting the Ethnic Central and South American criteria for WC.
Wang et al., 2019 [41]	Singapore	Randomized controlled trial	925 (50.8%)	64.7	WHtR, WC, BMI	Pre-diabetes or diabetes	Prediabetes was defined as meeting 1 of the 3 criteria: 1. Had FBG between 5.6 and <7.0 mmol/dL; 2. Had HbA1c levels between 5.7% and <6.5%. Diabetes was defined as: 1. Reported physician-diagnosed diabetes or taking antidiabetes medications; 2. Had FBG ≥7.0 mmol/dL; 3. HbA1c levels ≥6.5%. The thresholds of blood tests were based on 2018 ADA recommendations on diagnosis of prediabetes and diabetes.
Yang et al., 2018 [42]	China	Prospective cohort study	9962 (60.2%)	66.81 ± 5.55 (Non T2D cases), 66.44 ± 5.16 (Incident T2D cases)	WHtR, WC, BMI	T2DM	T2DM cases were defined according to the WHO criteria: 1. Self-reported physician-diagnosed diabetes or taking diabetes medications (oral hypoglycemic agent or insulin); Fasting glucose concentration ≥7.0 mmol/L.

*Abbreviations:* ADA, American Diabetes Association; BMI, body mass index; BP, blood pressure; DBP, diastolic blood pressure; FBG, fasting blood glucose; FPG, fasting plasma glucose; HbA1c, glycated hemoglobin; HDL-C, high-density lipoprotein cholesterol; IDF, International Diabetes Federation; JNC-7, Seventh Report of the Joint National Committee; LDL-C, low-density lipoprotein cholesterol; MetS, metabolic syndrome; NCEP-ATP III, National Cholesterol Education Program’s Adult Treatment Panel III; SBP, systolic blood pressure; TG, triglyceride; T2DM, type 2 diabetes mellitus; WC, waist circumference; WHO, World Health Organization; WHtR, waist-to-height ratio.

**TABLE 2:** Summary accuracy and the post-test probabilities of waist-to-height ratio, waist circumference and BMI screening for metabolic syndrome in older adults

Metabolic outcomes	Studies (*n*)	Sensitivity	Specificity	AUSROC	LR+	LR−	dOR	*I*_ZD_^2^	Cor
Metabolic syndrome									
WHtR									
Total	9	0.78 (0.69, 0.85)	0.69 (0.58, 0.77)	0.78 (0.78, 0.79)	2.48 (1.98, 3.09)	0.32 (0.25, 0.42)	7.65 (6.00, 9.75)	86.0%	0.90
Males	8	0.79 (0.73, 0.83)	0.67 (0.59, 0.74)	0.78 (0.77, 0.79)	2.35 (1.97, 2.79)	0.32 (0.28, 0.37)	7.29 (6.36, 8.36)	0.0%	1.0
Females	8	0.82 (0.71, 0.89)	0.62 (0.45, 0.76)	0.78 (0.77, 0.79)	2.13 (1.56, 2.91)	0.30 (0.22, 0.40)	7.22 (5.22, 9.98)	80.8%	0.92
WC									
Total	5	0.77 (0.63, 0.87)	0.63 (0.47, 0.76)	0.76 (0.74, 0.77)	2.08 (1.63, 2.64)	0.36 (0.27, 0.48)	5.77 (4.60, 7.25)	0%	0.82
BMI									
Total	7	0.78 (0.67, 0.86)	0.59 (0.47, 0.69)	0.74 (0.73, 0.74)	1.92 (1.60, 2.29)	0.37 (0.31, 0.44)	5.17 (4.75, 5.62)	43.1%	0.79
Males	6	0.81 (0.77, 0.84)	0.59 (0.54, 0.65)	0.77 (0.76, 0.79)	1.96 (1.70, 2.26)	0.34 (0.26, 0.44)	5.82 (3.99, 8.49)	14.9%	0.28
Females	6	0.81 (0.64, 0.92)	0.51 (0.31, 0.71)	0.73 (0.72, 0.74)	1.68 (1.29, 2.21)	0.36 (0.26, 0.48)	4.73 (3.82, 5.86)	43.1%	0.73
Hyperglycemia									
WHtR									
Total	5	0.76 (0.61, 0.87)	0.41 (0.26, 0.58)	0.62 (0.61, 0.63)	1.31 (1.16, 1.49)	0.55 (0.38, 0.79)	2.40 (1.58, 3.63)	69.9%	0.94
WC									
Total	5	0.66 (0.60, 0.72)	0.53 (0.49, 0.57)	0.61 (0.60, 0.61)	1.41 (1.30, 1.54)	0.63 (0.55, 0.73)	2.23 (1.79, 2.79)	48.7%	0.62

*Abbreviations:* Outcomes: BMI, body mass index; WC, waist circumference; WHtR, waist-to-height ratio; Measures: AUSROC, area under summary receiver operating characteristic curve for the discriminative power of a test; Cor, correlation between sensitivities and false positive rates; dOR, diagnostic odds ratio (the ratio of TP/FN to FP/TN) for general estimation of discriminative power of diagnostic procedures; *I*_ZD_^2^, *I*^2^ statistic to quantify the bivariate heterogeneity developed by Zhou and Dendukuri [43] for bivariate meta-analysis; LR+, positive likelihood ratio (the ratio of patients with the disease who test positive, to those who test positive but are disease free); LR−, negative likelihood ratio (the ratio of patients with the disease who test negative, compared to those who test negative and are disease free).

### MetS

**Figure 2:**
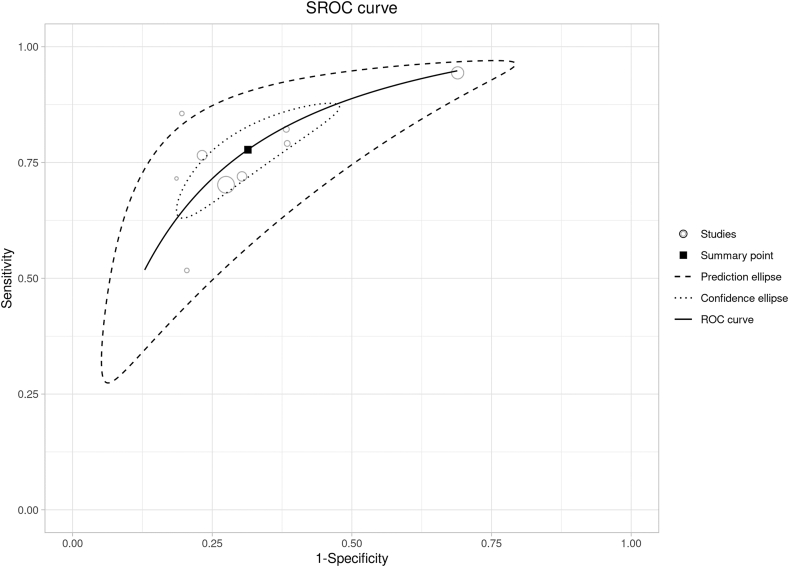
Summary receiver operating curve (SROC) of the diagnosis performance of WHtR for MetS in older adults. MetS, metabolic syndrome; WHtR, waist-to-height ratio.

Nine studies provided the optimal cutoffs of WHtR in predicting MetS [[26], [27], [28], [29],^31^,^36^,^37^,^39^,^40^]. The pooled results were outlined as follows (Table 2): AUSROC was 0.78 (95% CI: 0.78, 0.79), dOR was 7.65 (95% CI: 6.00, 9.75), sensitivity was 0.78 (95% CI: 0.69, 0.85), and specificity was 0.69 (95% CI: 0.58, 0.77). The SROC curve was shown in Figure 2; the solid circles represented the individual studies included in this meta-analysis, the solid curve summarized the overall diagnostic accuracy, and the dotted and dashed lines showed the confidence and prediction ellipses, respectively. Substantial heterogeneity (*I*^2^ = 86.0%) was observed.

Moreover, 5 studies provided the optimal cutoffs of WC in predicting MetS [26,27,29,36,39]. The pooled results were as follows: AUSROC was 0.76 (95% CI: 0.74, 0.77), dOR was 5.77 (95% CI: 4.60, 7.25), sensitivity was 0.77 (95% CI: 0.63, 0.87), and specificity was 0.63 (95% CI: 0.47, 0.76) (Supplemental Figure 1). No heterogeneity was reported (*I*^2^ = 0%).

Seven studies provided the optimal cutoffs of BMI in predicting MetS [[26], [27], [28], [29],^36^,^37^,^40^]. The pooled results were as follows: AUSROC was 0.74 (95% CI: 0.73, 0.74), dOR was 5.17 (95% CI: 4.75, 5.62), sensitivity was 0.78 (95% CI: 0.67, 0.86), and specificity was 0.59 (95% CI: 0.47, 0.69) (Supplemental Figure 2). Moderate heterogeneity (*I*^2^ = 43.1%) was observed. A higher dOR was observed for WHtR (7.65) in diagnosing MetS in older adults, compared with those for WC (5.77) and BMI (5.17).

**Figure 3:**
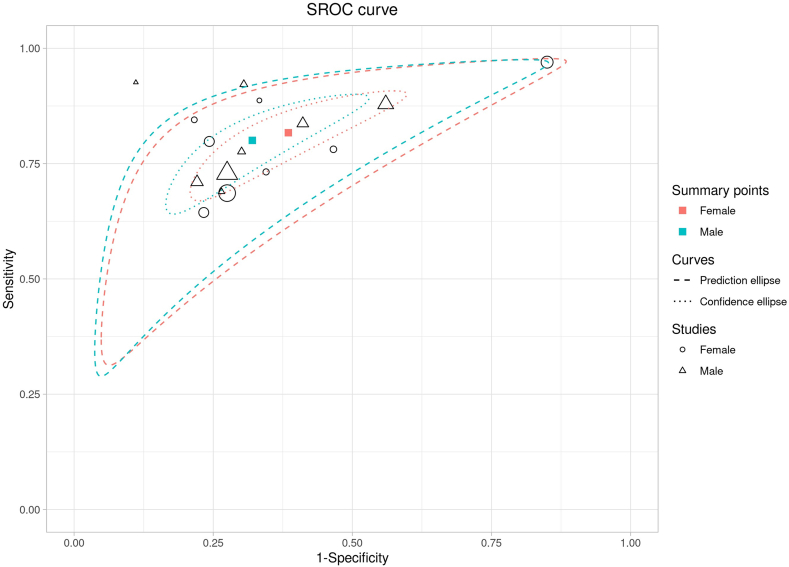
Summary receiver operating curve (SROC) of the diagnosis performance of WHtR for MetS in elderly males and females. MetS, metabolic syndrome; WHtR, waist-to-height ratio.

Subgroup analyses of WHtR and BMI by sex were also conducted. For WHtR, similar pooled results were observed in both males (AUSROC: 0.78; 95% CI: 0.77, 0.79; dOR: 7.29; 95% CI: 6.36, 8.36; sensitivity: 0.79; 95% CI: 0.73, 0.83; and specificity: 0.67; 95% CI: 0.59, 0.74) (Figure 3) and females (AUSROC: 0.78; 95% CI: 0.77, 0.79; dOR: 7.22; 95% CI: 5.22, 9.98; sensitivity: 0.82; 95% CI: 0.71, 0.89; and specificity: 0.62; 95% CI: 0.45, 0.76) (Figure 3). Substantial heterogeneity was observed in females (bivariate *I*^2^, *I*_Biv_^2^ = 80.8%) but not in males (*I*_Biv_^2^ = 0.0%). The SROC curves for males and females overlapped (Figure 3), and no significant difference was detected from the meta-regression (*P* = 0.641).

**Figure 4:**
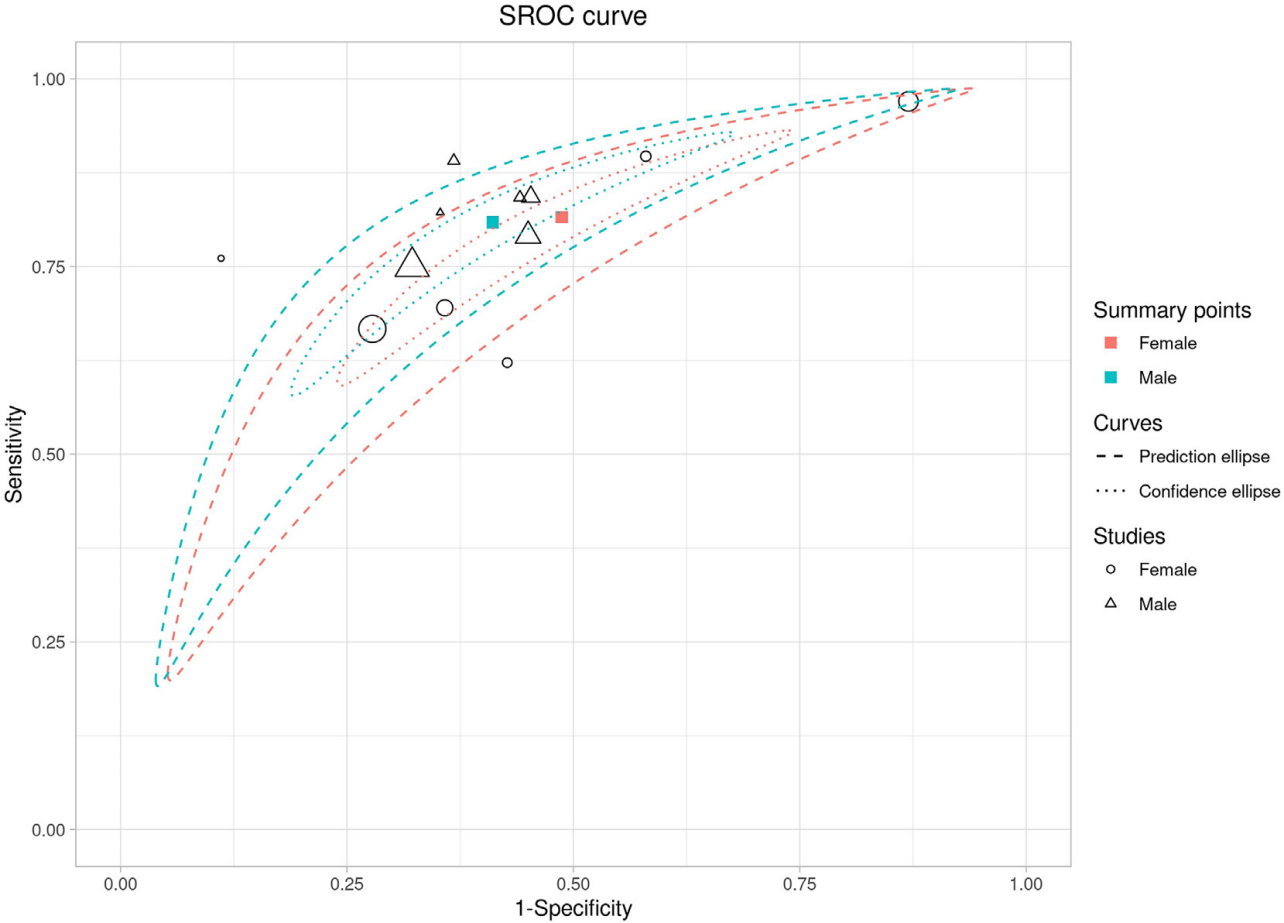
Summary receiver operating curve (SROC) of the diagnosis performance of BMI for MetS in elderly males and females. BMI, body mass index; MetS, metabolic syndrome; WHtR, waist-to-height ratio.

For BMI, comparable pooled results were observed in both males (AUSROC: 0.77; 95% CI: 0.76, 0.79; sensitivity: 0.81; 95% CI: 0.77, 0.84; and specificity: 0.59; 95% CI: 0.54, 0.65) (Figure 4) and females (AUSROC: 0.73; 95% CI: 0.72, 0.74; sensitivity: 0.81; 95% CI: 0.64, 0.92; and specificity: 0.51; 95% CI: 0.31, 0.71) (Figure 4). However, the pooled dOR of males was 5.82 (95% CI: 3.99, 8.49), which was higher than that of females (4.73; 95% CI: 3.82, 5.86). No moderate heterogeneity was detected in males (*I*^2^ = 14.9%), but it was in females (*I*^2^ = 43.1%). The SROC curves for males and females overlapped (see Figure 4), and no significant difference was detected from the meta-regression (*P* = 0.121).

### Hyperglycemia

Five studies provided the optimal cutoffs of WHtR in predicting hyperglycemia [27,33,34,41,42]. The pooled results were as follows: AUSROC was 0.62 (95% CI: 0.61, 0.63), dOR was 2.40 (95% CI: 1.58, 3.63), sensitivity was 0.76 (95% CI: 0.61, 0.87), and specificity was 0.41 (95% CI: 0.26, 0.58) (Supplemental Figure 3). Substantial heterogeneity was detected (*I*^2^ = 69.9%).

Moreover, 5 studies provided the optimal cutoffs of WC in predicting hyperglycemia [27,33,34,41,42]. The pooled results were as follows: AUSROC was 0.61 (95% CI: 0.60, 0.61), dOR was 2.23 (95% CI: 1.79, 2.79), sensitivity was 0.66 (95% CI: 0.60, 0.72), and specificity was 0.53 (95% CI: 0.49, 0.57) (Supplemental Figure 4). Moderate heterogeneity was observed (*I*^2^ = 48.7%). WHtR and WC had a comparable AUSROC (0.62 compared with 0.61) and dOR (2.40 compared with 2.23), while WHtR had a higher sensitivity (0.76 compared with 0.66) and lower specificity (0.41 compared with 0.53) on average. Due to the limited number of studies, subgroup analysis by sex was not performed.

### Hypertension

Two studies were not meta-analyzed and were presented narratively [30,38]. Jiang et al. [30] investigated the accuracy of BMI, WC, and WHtR in identifying hypertension in 14,364 Chinese participants aged ≥15 y. The accuracy indicator provided for the ≥65 y subgroup was inadequate for conducting meta-analysis. When comparing the AUCs of BMI, WC, and WHtR in males aged ≥65 y, BMI (AUC: 0.62; 95% CI: 0.58, 0.65) outperformed WHtR (AUC: 0.60; 95% CI: 0.57, 0.63) and WC (AUC: 0.59; 95% CI: 0.55, 0.62) in diagnosing hypertension. For females aged ≥65 y, BMI (AUC: 0.60; 95% CI: 0.57, 0.63) and WHtR (AUC: 0.61; 95% CI: 0.58, 0.63) were comparable in diagnosing hypertension, while WC (AUC: 0.59; 95% CI: 0.56, 0.62) had lower discriminative power.

Nguyen Ngoc et al. [38] evaluated the accuracy of BMI, WC, and WHtR in discriminating hypertension in 15,842 participants with a mean age of 59.3 ± 13.2 y in Thailand. Similarly, the accuracy indicators provided were insufficient to perform meta-analysis. Overall, WHtR (AUC: 0.64; 95% CI: 0.63, 0.65; 57.6% sensitivity; 63.2% specificity) had the strongest ability to discriminate hypertension in middle-aged and older adults of both sexes, followed by WC (AUC: 0.63; 95% CI: 0.62, 0.64; 57.7% sensitivity; 62.8% specificity) and BMI (AUC: 0.60; 95% CI: 0.59, 0.61; 55.2% sensitivity; 60.2% specificity). The optimal cutoff of WHtR for diagnosing hypertension in both males and females was 0.52.

For the remaining 3 studies [27,32,34], although accuracy indicators were provided, at least 5 studies were required for SROC analysis. Therefore, they were presented narratively. Liu et al. [34] studied the accuracy of BMI, WC, and WHtR in identifying hypertension in 4416 Chinese participants aged ≥65 y. For older people, BMI had a superior ability in identifying hypertension (AUC: 0.70; 95% CI: 0.67, 0.72; 64% sensitivity; 41% specificity), followed by WHtR (AUC: 0.67; 95% CI: 0.65, 0.70; 72% sensitivity; 44% specificity), and WC (AUC: 0.67; 95% CI: 0.64, 0.69; 72% sensitivity; 46% specificity). The optimal cutoff of BMI for identifying hypertension was 23.60 kg/m^2^.

Kawamoto et al. [32] investigated the accuracy of WHtR in predicting hypertension in 1727 Japanese participants with an average age of 70 y. The results suggested that WHtR had significant predictive ability in identifying hypertension in males (AUC: 0.65; 95% CI: 0.61, 0.69; 44.3% sensitivity; 80.2% specificity) and females (AUC: 0.69; 95% CI: 0.66, 0.735; 61.0% sensitivity; 68.6% specificity). The optimal cutoffs of WHtR for identifying hypertension in males and females were 0.53 and 0.54, respectively.

Gharipour et al. [27] evaluated the accuracy of WC, BMI, and WHtR in identifying hypertension in 206 male Iranians aged >65 y. The results suggested that BMI had a better ability to distinguish hypertension (AUC: 0.64; 95% CI: 0.54, 0.74; 82% sensitivity; 49% specificity) than WC (AUC: 0.63; 95% CI: 0.54, 0.72; 75% sensitivity; 49% specificity) and WHtR (AUC: 0.62; 95% CI: 0.53, 0.71; 76% sensitivity; 54% specificity]. The optimal BMI cutoff for identifying hypertension in males was 22.84 kg/m^2^.

### Dyslipidemia

Three studies examined the ability of BMI, WC, and WHtR in identifying dyslipidemia [27,34,35]. They were presented narratively as at least 5 studies were required to perform SROC analysis. Liu et al. [34] studied the accuracy of BMI, WC, and WHtR in identifying dyslipidemia in 4416 Chinese participants aged ≥65 y. The results showed that WHtR had the highest accuracy (AUC: 0.62; 95% CI: 0.60, 0.63; 68% sensitivity; 50% specificity) in diagnosing dyslipidemia among all parameters, followed by BMI (AUC: 0.61; 95% CI: 0.59, 0.63; 68% sensitivity; 50% specificity) and WC (AUC: 0.59; 95% CI: 0.57, 0.61; 76% sensitivity; 61% specificity). The optimal WHtR cutoff for diagnosing dyslipidemia in older adults was 0.51.

Vidal Martins et al. [35] evaluated the abilities of BMI, WHtR, and WC at the midpoint between the last rib and the iliac crest (WC_1_), the minimal WC (WC_2_) and umbilical WC (WC_3_) in predicting dyslipidemia in 349 older patients in Brazil. In males, all anthropometric indicators showed comparable abilities in predicting dyslipidemia. Among them, WC_2_ had the best discriminative power (AUC: 0.74; 70.2% sensitivity; 64.3% specificity). The optimal cutoff point of WC_2_ for predicting dyslipidemia in older males was 88 cm. Similarly, in females, all included anthropometric variables showed comparable abilities in predicting dyslipidemia. Among them, BMI had the best discriminative power (AUC: 0.65; 61.4% sensitivity; 59.4% specificity). The optimal cutoff point of BMI for predicting dyslipidemia in older females was 27.8 kg/m^2^.

Gharipour et al. [27] investigated the accuracy of WC, BMI, and WHtR in identifying dyslipidemia in 206 male Iranians aged >65 y. WC demonstrated a better ability in predicting high triglycerides (AUC: 0.63; 95% CI: 0.55, 0.71; 67% sensitivity; 59% specificity) and low HDL (AUC: 0.61; 95% CI: 0.53, 0.69; 63% sensitivity; 58% specificity) among all parameters. The optimal cutoff points of WC for predicting high triglycerides and low HDL in older males were both 94.5 cm.

### Quality assessment of the included studies

Quality assessment of the included studies was conducted using QUADAS-2, and the results were shown in Table 3. In terms of risk of bias, 14 studies (82.4%) [[26], [27], [28], [29], [30],[32], [33], [34], [35], [36], [37], [38], [39], [40]] had no or only one domain judged as high risk. In terms of applicability concerns, only 2 studies (11.8%) [39,41] had one domain judged as high risk, while the other 15 studies (88.3%) [[26], [27], [28], [29], [30], [31], [32], [33], [34], [35], [36], [37], [38],^40^,^41^] were judged as low risk in all domains.

**TABLE 3:**
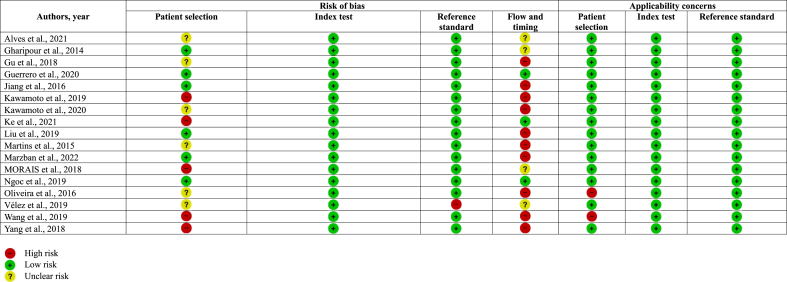
Quality assessment of included studies.

For patient selection, the risks of bias in 5 studies (29.4%) [31,33,37,41,42] were high because a random sample of patients was not recruited and that of 6 studies (35.3%) [26,28,32,35,39,40] were rated as unclear because sampling details in recruitment were not mentioned. The risks of bias of all studies were judged as low risk in index test domains. For reference standard, risk of bias was rated as high in one study (5.9%) [40] because the cardiometabolic risk index was self-calculated. For flow and timing, the risks of bias were high in 10 studies (58.8%) [28,[30], [31], [32],[34], [35], [36],^39^,^41^,^42^] because not all participants were included in the analysis or received a reference standard, while that of 4 studies (23.5%) [26,27,37,40] were unclear.

In terms of applicability concerns, 2 studies (11.8%) [39,41] were judged as high risk in the patient selection domain because one only included patients with hypertension [41] and one included patients residing in institutions [39]. In both the index test and reference standard domains, all studies had low applicability concerns.

## Discussion

### Summary of main findings

In the present meta-analysis, we pooled the diagnostic test accuracy of WHtR, BMI, and WC to screen for MetS and its components among older adults aged ≥65. Despite a range of optimal cutoff values, WHtR performed better than BMI and WC in screening for MetS among older adults aged ≥65, as reflected by the higher dOR and is potentially better among the male population. For hyperglycemia, the performances of WHtR and WC were comparable. Despite having inadequate data for meta-analysis, studies that included elevated blood pressure and dyslipidemia as outcomes also showed similar diagnostic performances of all included adiposity indicators.

### Comparing the screening power of adiposity indicators

As shown in meta-analysis, while the pooled sensitivity was comparable for WHtR to screen for MetS and hyperglycemia, higher pooled specificity, AUSROC, and dOR were observed when using WHtR to screen for MetS than hyperglycemia, and the observation was similar when using WC as an indicator. Although the definition of MetS varies, central obesity as defined by WC has been one of the criteria for most definitions, which suggests elevated WHtR and WC may be more closely related to MetS than hyperglycemia. Furthermore, when comparing WHtR and WC to screen for MetS, the pooled sensitivity, specificity, and AUSROC were comparable, but WHtR had a higher dOR. While this finding may demonstrate a relative advantage of WHtR over WC to screen for MetS, the difference was not substantial. It is probable that older adults are being classified with elevated WHtR due to height loss, hence affecting the accuracy of WHtR. There is a need to identify the appropriate WHtR cutoff for older adults, and more studies that examine the accuracy using different cutoff values for more comprehensive research evidence are needed. Furthermore, when comparing the diagnostic accuracy of WHtR to screen for MetS in younger populations, a meta-analysis that included children and adolescents found dOR values higher than those in the present study (7.65) when the cutoff values were 0.46 to 0.50 (11.37) or ≥0.51 (32.46) [17], while the cutoff values of our included studies were no lower than 0.49. It is possible that general obesity and central obesity have differential effects on the health risk of older people when compared to the younger population [44], but because of the huge differences in population characteristics, this observation is hypothesis-generating and warrants examination in future studies.

### Reasons for sex differences in screening power

Another interesting observation is that WHtR (7.29 compared with 7.22) and BMI (5.82 compared with 4.73) had higher dORs when screening for MetS among older male adults than their female counterparts, reflecting a potential sex difference in screening power for both indicators of general (BMI) and central (WHtR) obesity. One possible reason is the differences in fat distribution between males and females, especially after menopause. The hormonal shifts that occur during midlife when females have a higher androgen to estradiol ratio after menopause have been linked to the increase in total and central fat [45]. This physiological change may mask the elevation in WHtR and BMI due to unhealthy diet and physical inactivity, hence misclassifying the cardiometabolic risk of postmenopausal females. However, it is unclear whether the sex difference is also consistent when using WC and/or BMI for other components of MetS, which warrants confirmation from further studies.

### Quality of the included studies and the validity of the study findings

In terms of study quality as assessed by QUADAS-2, risk of bias and applicability concerns were low in general, except for “patient selection” and “flow and timing” for risk of bias; less than half of the studies had low risk of bias. For “patient selection,” the most common reasons for unclear and high risk of bias were missing information of the sampling plan (6 studies) and may have a selected group of participants (5 studies). For “flow and timing,” the most common reasons for unclear and high risk of bias were having no information about drop-out (4 studies) and not including all participants in the analysis (10 studies). These issues affected the representativeness of study populations and should be acknowledged as limitations of the included studies. In terms of the measurement of exposures (WHtR, WC, and BMI) and outcomes (MetS and its components), as well as the analysis of screening power, most included studies performed assessment and analysis in standardized manner, and therefore, there is less concern on the validity of findings.

### Strengths and limitations

The strengths of the present study included compliance with PRISMA guidelines and the robust statistical approach to summarize the diagnostic test accuracy of WHtR, BMI, and WC using the pooled sensitivity and specificity, estimation of likelihood ratios and dORs, and generating SROC curves. However, there are limitations that readers should be aware of. Because of the limited number of available studies with data on older adults, the present study was not able to identify the optimal cutoff values of WHtR in screening for MetS, although the pooled sensitivity and specificity appeared to be acceptable regardless of cutoff values. Furthermore, all included studies were cross-sectional in nature, which might make them subject to reverse causation bias. Moreover, Meta-DiSc 2.0 assumed the variances of the subgroups (i.e., sex for the present study) were the same, but the estimated variance in each subgroup was not provided. Therefore, we used the “mada” package in R to compute the variances of each subgroup for comparison (WHtR: τ_M_^2^=0.227 and τ^F^_2_=0.262; BMI: τ_M_^2^=0.029 and τ^F^_2_=0.051), and no significant difference was observed, which verifies the assumption of Meta-DiSc 2.0. Another notable but generic limitation for meta-analysis is that the bivariate *I*^2^ statistic depends on the sample size; therefore, the *I*^2^ values among meta-analyses with different numbers of included studies may not be comparable. In addition, it is known that threshold effect might affect the results because studies might have different thresholds or criteria that greatly influence the estimation of summary points. For examining the potential threshold effect, we added the correlation between the sensitivities and FP rates in Table 2, for which a high positive correlation coefficient (i.e., *r* ≥ 0.6 for correlation analysis between sensitivity and FP rate) was observed [46], suggesting the threshold effect. To address this limitation, Meta-DiSc 2.0 deals with the threshold effect using the bivariate model. However, we cannot fully rule out the impact of the threshold effect in our results and require cautious interpretation of the findings. Last but not least, the outcome definition varied across studies, e.g., using the National Cholesterol Education Program’s Adult Treatment Panel III definition, the harmonized definition, the International Diabetes Federation Joint Interim Statement, etc., for classifying MetS, which can be another source of heterogeneity. To enhance comparability across studies, future studies may report the results using multiple outcome definitions so that the more commonly used definition can be selected for meta-analysis.

In conclusion, WHtR had better performance than BMI and WC in screening for MetS among older adults aged ≥65, as reflected by the higher dOR, and was potentially better among the male population. However, studies on the diagnostic test accuracy of adiposity indicators and components of MetS are still inadequate. Future studies should focus on older adults, or include older adults as a subgroup of the participants, to help determine the cutoff values of WHtR in diverse populations.

## Author contributions

The authors’ responsibilities were as follows—KL, MW, WT: designed research; LC, VC: conducted research; KL, LC, MW, VC, WT: analyzed data; KL, LC, MW, VC, WT: wrote the paper; KL, WT: had primary responsibility for final content; and all authors: read and approved the final manuscript.

## Conflict of interest

The authors report no conflicts of interest.

## Funding

The research was supported by Start-up Fund for New Recruits (Grant number: BE91).

## Data availability

Data described in the manuscript, code book, and analytic code will be made available upon request pending.
