# Supplementary material for: Diagnostic Accuracy of Waist-to-Height Ratio, Waist Circumference, and Body Mass Index in Identifying Metabolic Syndrome and Its Components in Older Adults: A Systematic Review and Meta-Analysis
Source: Curr Dev Nutr. 2023 Dec 12;8(1):102061. doi: 10.1016/j.cdnut.2023.102061 (PMC10790020; doi:10.1016/j.cdnut.2023.102061)
Supplement: Multimedia component 1 [file mmc1.docx]

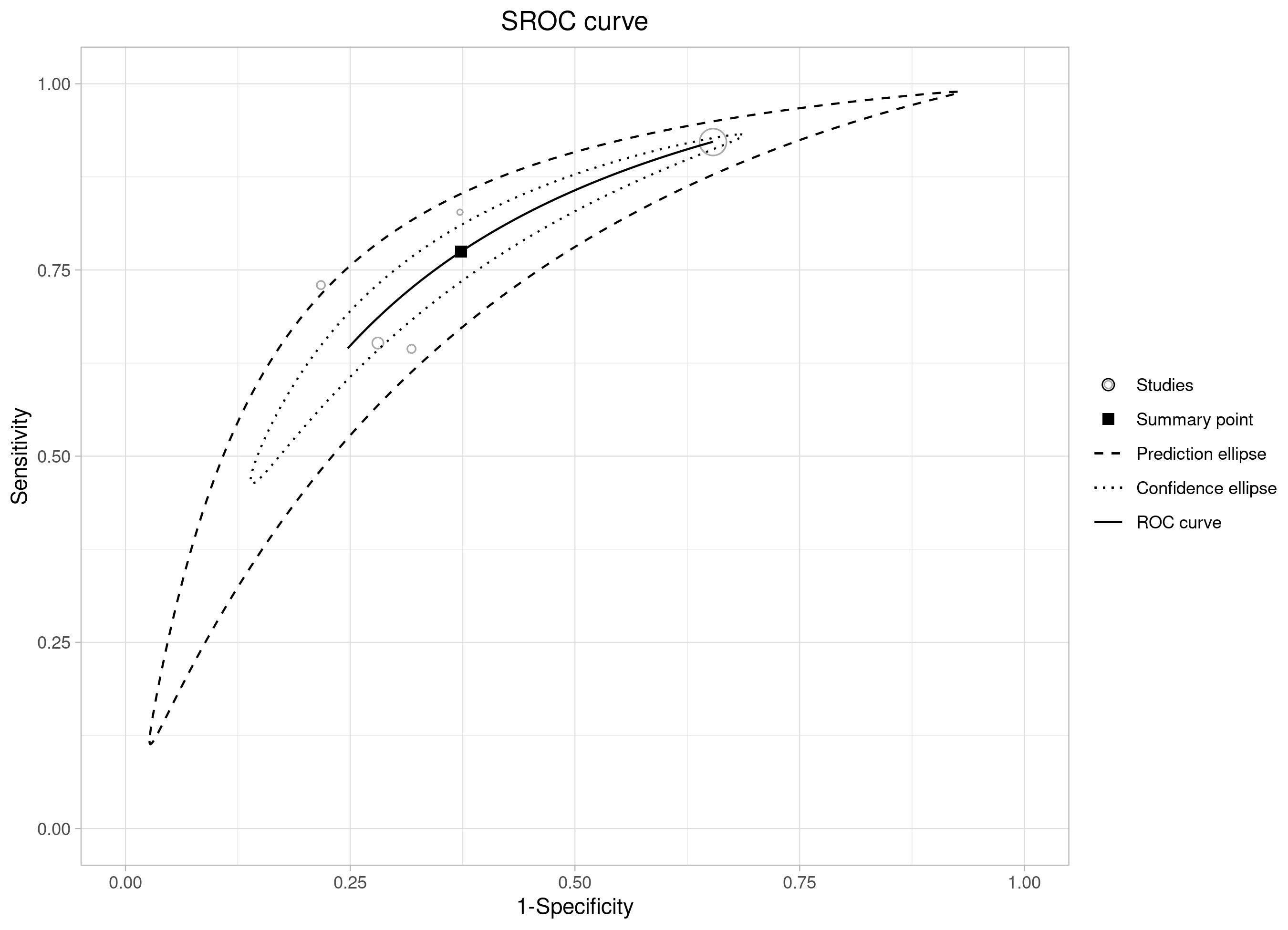


**Figure S1.** Summary receiver operating curve (SROC) of the diagnosis performance of WC for MetS in older adults.
