# Supplementary material for: Diagnostic Accuracy of Waist-to-Height Ratio, Waist Circumference, and Body Mass Index in Identifying Metabolic Syndrome and Its Components in Older Adults: A Systematic Review and Meta-Analysis
Source: Curr Dev Nutr. 2023 Dec 12;8(1):102061. doi: 10.1016/j.cdnut.2023.102061 (PMC10790020; doi:10.1016/j.cdnut.2023.102061)
Supplement: Multimedia component 5 [file mmc5.docx]

**Table S1.** Optimal cut-off values of waist-to-height ratio (WHtR) of studies included in meta-analysis.

| Authors, Year | WHtR optimal cut-off values | | | | | |
| --- | --- | --- | --- | --- | --- | --- |
|  | **Metabolic Syndrome** | | | **Hyperglycemia** | | |
|  | **Total** | **Male** | **Female** | **Total** | **Male** | **Female** |
| Alves et al., 2021 | 0.58 | 0.57 | 0.58 | -- | -- | -- |
| Gharipour et al., 2014 | 0.59 | -- | -- | 0.56 | -- | -- |
| Gu et al., 2018 | -- | 0.51 | 0.52 | -- | -- | -- |
| Guerrero et al., 2020 | 0.62 | 0.64 | 0.62 | -- | -- | -- |
| Kawamoto et al., 2019 | -- | 0.52 | 0.53 | -- | -- | -- |
| Ke et al., 2021 | -- | -- | -- | -- | 0.49 | 0.52 |
| Liu et al., 2019 | -- | -- | -- | 0.50 | -- | -- |
| Marzban et al., 2022 | 0.54 | 0.55 | 0.53 | -- | -- | -- |
| MORAIS et al., 2018 | -- | 0.55 | 0.59 | -- | -- | -- |
| Oliveira et al., 2016 | 0.55 | 0.56 | 0.55 | -- | -- | -- |
| Vélez et al., 2019 | -- | 0.56 | 0.63 | -- | -- | -- |
| Wang et al., 2019 | -- | -- | -- | -- | 0.54 | 0.55 |
| Yang et al., 2018 | -- | -- | -- | -- | 0.51 | 0.51 |
